# Chemically defined 3D matrix for *in vitro* maturation (IVM) of human oocytes

**DOI:** 10.5935/1518-0557.20250020

**Published:** 2025

**Authors:** Adriana Bos-Mikich, Gabriella M. Andrade, Luis Alberto L. dos Santos, Nilo Frantz

**Affiliations:** 1 Universidade Federal do Rio Grande do Sul, Porto Alegre, RS, Brazil; 2 Nilo Frantz Reproductive Medicine, Porto Alegre, RS, Brazil

**Keywords:** 3D matrix, rescue IVM, PVA

## Abstract

**Objective:**

Improvements in oocyte culture conditions may enhance oocyte maturation rates. PVA has been used in tissue engineering scaffolds to provide suitable strength and adhesive properties to support cell adhesion and proliferation. The aim of the present study was to test a three-dimensional PVA matrix for human rescue oocyte maturation.

**Methods:**

Immature human oocytes and cumulus cells were obtained from patients undergoing conventional IVF cycles. Firstly, two replicates of cumulus cells exposed to PVA-BTCA matrices were performed to assess a possible cytotoxic effect of the matrix. Next, a total of 23 immature oocytes and respective cumulus cells were split between the control and PVA-BTCA-containing culture system for rescue IVM, at 38oC and 5%CO_2_.

**Results:**

Cumulus cells exposure to PVA matrix allows for cell survival and adhesion to the substrate, confirming its non-toxicity. The overall maturation rate in the PVA-BTCA culture system was 66.6% (n=10/15): 77.7% of VG (n=7/9) and 50% (n=3/6) of MI oocytes reached MII. The control culture received 8 VG oocytes, of which 37.5% (n=3) reached MI and 62.5% reached MII (n=5).

**Conclusions:**

PVA-BTCA-containing culture system may represent an alternative for human oocyte maturation procedures.

## INTRODUCTION

Human rescue in vitro maturation (rIVM) represents an interesting procedure to be employed in assisted reproduction cycles, whenever superovulation resulted in the collection of a small number of mature oocytes and/or when in vivo maturation rate was not satisfactory and there is a significant proportion of immature gametes at oocyte pick up. It is estimated that 15-20% of oocytes collected in IVF cycles are immature (Smitz *et al*., 2004), and they usually are left in culture to finalize meiosis I and reach metaphase II. However, these oocytes were denuded for maturation check and culture conditions may not be ideal for the completion of nuclear and cytoplasmic maturation in vitro, resulting in unsatisfactory maturation rates; posing the question on whether it is worthwhile to perform this additional procedure in a busy IVF lab. Another point to be taken into consideration is the fact that immature oocytes submitted to rIVM may be used as a source of MII oocytes to be used for stem cell research, after artificial stimulation, to generate haploid or diploid parthenogenetic stem cells. Last, future studies on IVM for Polycystic Ovarian Syndrome (POS) patients and fertility preservation for young pre-pubertal girls may benefit from IVM systems developed on immature oocytes obtained from stimulated ovaries.

The presence of cumulus cells and follicular structure play an important role in the *in vivo* maturation process, as oocytes need cumulus cells to support their growth and to accumulate cytoplasmic components, essential for subsequent fertilization and embryonic development (Russell *et al*., 2016; Sugimura *et al*., 2018). Most, if not all rIVM are performed in IVF culture medium in a 2D system, in absence of follicular cells. This ordinary IVM culture conditions may be related to the lower performance of rescued MII oocytes in terms of fertilization, embryo development and live birth outcomes compared to results obtained from in vivo matured MII oocytes (De Vos *et al*., 2016; Galvão *et al*., 2018). Thus, the possibility to mimic the follicular environment in a 3D *in vitro* system may improve rIVM results and propel their use for infertility treatments, fertility preservation and stem cell research.

The aim of the present study was to assess the maturation rates of immature germinal vesicle (GV) and metaphase I (MI) oocytes collected from stimulated ovaries and cultured in a 3D poly-vinyl alcohol matrix (PVA), in the presence of loose cumulus cells.

## MATERIALS AND METHODS

### Patients

Infertility treatment patients older than 18 years of age, invited to participate in the project were included in the study after signing a consenting form. This study project was approved by the ethics committee of the Federal University of Rio Grande do Sul (CAEE no. 50587321.6.0000.5327). Controlled ovarian stimulation protocols with GnRH antagonist suppression, recombinant FSH stimulation, and trigger (GnRH agonist and/or hCG) were personalized according to patient´s characteristics such as age, AMH, FSH and LH serum levels. Transvaginal oocyte collection was performed 36 hours post-trigger administration.

### PVA-BTCA matrices generation

Three grams of polyvinyl alcohol (PVA, Mw 130,000, 99% degree of hydrolysis, Sigma Chemical Co., St. Louis, MO) were dissolved in 30 ml of boiling water under stirring. After dissolving the PVA, temperature was lowered to 60^o^C and 0.3ml of acetic acid together with 0.3grams of BTCA (1,2,3,4-Butane tetracarboxylic acid; Sigma Chemical Co., St. Louis, MO, 99% batch MKCM9767) were added to the system and kept under stirring for further 20 minutes. Once cooled, the solution was placed in a petri dish (9 cm diameter) and left for polymerization in an incubator at 60^o^C for 24 hrs. The obtained membrane was cut into disks of 15mm diameter, which were placed in distilled deionized water for swelling. The swollen matrices were transferred to an incubator at 60^o^C, for 24 hours for drying and stored in plastic bags until use. On the day before cultures, the matrices were sterilized by UV light for 10 minutes, washed twice in PBS solution, covered with CSC culture medium supplemented with 10% de SSS and left in a CO_2_ incubator at 37^o^C for a minimum of 24 hours for hydration. After this period, cumulus cells only or cumulus cells and oocytes were added to the culture system, according to the experimental groups.

### Cumulus oocyte complex (COCs) collection and assessment

Follicle aspiration was performed with phosphate buffered solution (PBS) supplemented with heparin at 37^o^C. Oocyte cumulus cells complexes were removed from the follicular fluid and transferred to MHM (Multipurpose Handling Medium - IrvineScientific®) supplemented with 10% SSS (Serum Substitute Supplement - IrvineScientific®) for 4 minutes and then transferred to CSC (Continuous Single Culture - IrvineScientific®) supplemented with 10% SSS for 3h, before denudation with hialuronidase (IrvineScientific®) and a stripper pipette. Oocytes were classified as mature MII, immature MI or GV. Only MI and GV oocytes were used for rIVM experiments.

Discarded cumulus cells were collected, centrifuged twice at 200g for 5 minutes and the pellet was suspended in fresh CSC medium supplemented with 10% SSS for counting in a Neubauer chamber.

### Cumulus cells culture in PVA-BTCA matrix

The first set of experiments assessed the putative cytotoxicity of the PVA-BTCA matrices. After counting, approximately 5x10^5^cumulus cells (Andrade *et al*., 2017; Portela *et al*., 2010; Gutierrez *et al*., 1997) were placed in four 4-well culture dishes containing one PVA-BTCA matrix embedded in 500µL of CSC and left in culture at 37^o^C, 5% CO_2_, for 24-48hrs.

### rIVM

Two experimental groups were formed for rIVM:

1. rIVM-Matrix: oocytes cultured in PVA-BTCA matrices together with cumulus cells;

2. rIVM-Control: oocytes cultured without PVA-BTCA matrices, with cumulus cells.

Before being used for culture, PVA-BTCA matrices were sterilized by UV light (10 minutes) and maintained in CSC medium overnight for hydration. Cumulus cells and immature oocytes were split in culture dishes according to the experimental groups, for 24-30 h culture at 37^o^C and 5%CO_2_.

## RESULTS

**Figure 1:**
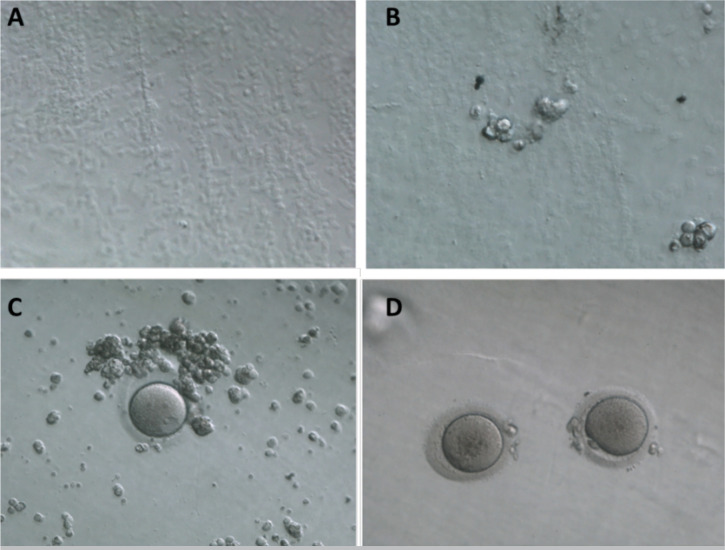
Illustration of PVA-BTCA matrix and rescue *in vitro* maturation (IVM) of human oocytes study experimental groups. A. PVA-BTCA matrix surface under the microscope (10x magnification); B. Presence of cumulus cells adhered to the matrix; C. Oocyte in germinal vesicle (VG) under the PVA matrix and D. Oocytes matured in the 3D environment (PVA-BTCA matrix) in co-culture with cumulus cells.

Seven patients participated in the experiments: two donated cumulus cells for the cytotoxicity tests and five donated cumulus cells and immature oocytes for the rIVM experiments (Figure 1).

### Cumulus cells exposure to PVA-BTCA matrices

Cumulus cells were used for the PVA-BTCA matrix cytotoxicity test, in two experimental repeats. In both occasions, we observed cells attached to the matrices suggesting no deleterious effect of the PVA-BTCA matrices on cell viability, after 24 or 48 h (Figure 1, A and B).

### Oocyte-cumulus cell co-culture

The rIVM - matrix culture system received nine VG and six MI oocytes in five experimental replicates. The overall maturation rate was 66.6% (n=10/15): 77.7% of VG (n=7/9) and 50% (n=3/6) of MI oocytes reached MII. The control culture without PVA matrix received eight VG oocytes, of which 37.5% (n=3) reached MI and 62.5% reached MII (n=5) (Table 1). These results showed that PVA-BTCA matrices are not toxic for cumulus cells and oocytes, and represent an interesting 3D culture system for rIVM, without any deleterious effect on both, cumulus cells and oocytes (Figure 1, B, C and D).

**Table 1:** Results of *in vitro* rescue maturation experiments.

Replicates	Control		PVA-BTCA matrix	
	Initial oocyte stage	Final oocyte stage	Initial oocyte stage	Final oocyte stage
**1**	-	-	2 MI	1 MI e 1 MII
**2**	2 VG	1 MI e 1 MII	2 VG	2 MII
**3**	1 VG	1 MII	1 VG	1 VG
**4**	1 VG	1 MII	1 VG	1 MII
**5**	4 VG	2 MI e 2 MII	5 VG	1 MI e 4 MII
			4 MI	2 MI e 2MII

## DISCUSSION

Present results showed that PVA-BTCA matrix is a non-toxic 3D scaffold for use in human rescue IVM. Despite the low number of immature oocytes obtained for rIVM, the overall maturation rate in the matrix culture system was superior to that in 2D culture system, where the cells and oocytes remain loose in the culture dish.

Why use a 3D culture system for IVM? Hypothetically, the immature oocyte and cumulus cells removed from the ovarian follicles would perform better cytoplasmic and nuclear maturation in an environment that mimics the ovarian follicle structure. Thus, different biological or chemically defined materials have been tested to build 3D systems for human IVM (Crocco *et al*., 2013), in order to facilitate cell adhesion and cell-to-cell communication, fundamental processes for cell viability, growth and differentiation (Zhao *et al*., 2014; Chavoshinezhad & Niknafs, 2024). Synthetic, chemically defined materials present the advantage of a defined composition, which does not vary from one production batch to the next. The units are manufactured on the same production line, using the same techniques and components. PVA-BTCA matrices have been used in protocols for stem cell generation, showing derivation and new colony formation from bovine parthenogenetic embryos and loose iPS cells (Ruggeri *et al*., 2012; Sanguinet *et al*., 2020).

It is well known that immature oocytes collected in stimulated cycles are able to resume meiosis *in vitro* and give rise to live birth after fertilization and embryo development. However, *in vitro* maturation of oocytes collected from small antral follicles and live birth rates are still well below that of oocytes collected from *in vivo* matured oocytes, suggesting that *in vitro* culture conditions are not satisfactory to promote full ooplasmic and nuclear maturation. Maintaining oocyte/cumulus cells in close proximity in a 3D environment may facilitate the correct interchange of molecules and factors between the two cell types, providing a more physiological way to acquire fertilization, and embryonic/fetal development competence.

The results of the present study will allow us to further apply 3D PVA-BTCA matrices for in vitro maturation of human oocytes collected from non-stimulated SOP ovaries and /or ovarian fragments collected from patients undergoing fertility preservation for oncological disorders.

## CONCLUSION

PVA-BTCA matrices may represent an inexpensive and easy to obtain substrate to be added to *in vitro* maturation systems, to promote adequate follicle/oocyte growth and maturation.
